# *JAZF1–SUZ12* endometrial stromal sarcoma forming subserosal masses with extraordinary uptake of fluorodeoxyglucose on positron emission tomography: a case report

**DOI:** 10.1186/s13000-019-0897-y

**Published:** 2019-10-15

**Authors:** Koto Fujiishi, Shigenori Nagata, Rieko Kano, Chiaki Kubo, Maasa Shirayanagi, Megumi Ozaki, Takashi Yamamoto, Katsuyuki Nakanishi, Shoji Kamiura, Shin-ichi Nakatsuka

**Affiliations:** 1 Department of Diagnostic Pathology and Cytology, Osaka International Cancer Institute, Osaka, Japan; 2 Department of Clinical Laboratory, Osaka International Cancer Institute, Osaka, Japan; 3 Department of Diagnostic and Interventional Radiology, Osaka International Cancer Institute, Osaka, Japan; 4 Department of Gynecologic Oncology, Osaka International Cancer Institute, Osaka, Japan

**Keywords:** Endometrial stromal sarcoma, *JAZF1–SUZ12*, Subserosal mass, Cyclin D1, Positron emission tomography

## Abstract

**Background:**

Low-grade endometrial stromal sarcoma (ESS) is rare mesenchymal neoplasm, recently specified as harboring *JAZF1–SUZ12* rearrangement. Typical *JAZF1–SUZ12* ESS is slow growing, in which high uptake of fluorodeoxyglucose (FDG) on positron emission tomography (PET) and subserosal masses are quite unusual.

**Case presentation:**

A 69-year-old Japanese woman complained of urinary incontinence. Pelvic magnetic resonance imaging showed uterine lesions composed of (1) a 9 × 8 × 7-cm mass protruding from the right-anterior wall, (2) a 4.5-cm mass attached to the right-posterior wall, and (3) a 6.5-cm intramural mass in the fundus. FDG-PET demonstrated maximum standardized uptake value of 13.28 confined to the two subserosal masses (1 & 2) in contrast to no uptake of the intramural mass (3). She was diagnosed with a high-grade uterine sarcoma concomitant with leiomyomas and underwent total hysterectomy with bilateral salpingo-oophorectomy and pelvic lymphadenectomy. The removed uterus had three tumors—two in the right-anterior and right-posterior subserosa, respectively, and the remaining in the fundal myometrium. Microscopically, the three tumors shared morphologic features characterized by neoplastic cells similar to proliferative-phase endometrial stromal cells, in which neither round-cell component, pleomorphism, nor high mitotic activity was recognized. Nuclear cyclin D1 immunostaining was identified 50% of neoplastic cells in the two subserosal tumors (1 &2) whereas < 1% positive cells in the intramural component (3). Reverse transcriptase-polymerase chain reaction showed the same-sized electrophoretic bands indicating *JAZF1–SUZ12* gene fusion shared by the three uterine tumors and a focal tumor extension into the extrauterine vein. The patient is alive without evidence of recurrence at 14 months after surgery.

**Conclusions:**

Pathologists and clinicians should not exclude the possibility of *JAZF1–SUZ12* ESS even when uterine subserosal masses demonstrate extraordinary FDG uptake on PET. Molecular analysis is helpful for diagnostic confirmation of *JAZF1–SUZ12* ESS with a complex growth pattern.

## Background

Endometrial stromal tumors are uncommon; they account for less than 10% of uterine mesenchymal neoplasms and less than 2% of all uterine tumors [1]. They are sorted by the 2014 World Health Organization (WHO) classification into four categories: endometrial stromal nodule, low-grade endometrial stromal sarcoma (ESS), high-grade ESS, and undifferentiated uterine sarcoma [2]. Low-grade ESS is the second most common mesenchymal malignancy of the uterus and recently characterized by harboring the genetic rearrangement t (7;17)(p15;q21) resulting in *JAZF1–SUZ12* gene fusion [3]. Low-grade ESS is typically slow growing, in which high uptake of fluorodeoxyglucose (FDG) on positron emission tomography (PET) and subserosal masses are much less common than in leiomyosarcomas. Here, we describe a woman with *JAZF1–SUZ12* ESS of the uterine corpus forming two sizeable subserosal masses other than a conventional intramural component, the two former of which exhibited exceptionally high FDG uptake on PET/computed tomography indicating distinctive heterogeneity in proliferative activity within a single tumor.

## Case presentation

### Clinical course

**Fig. 1:**
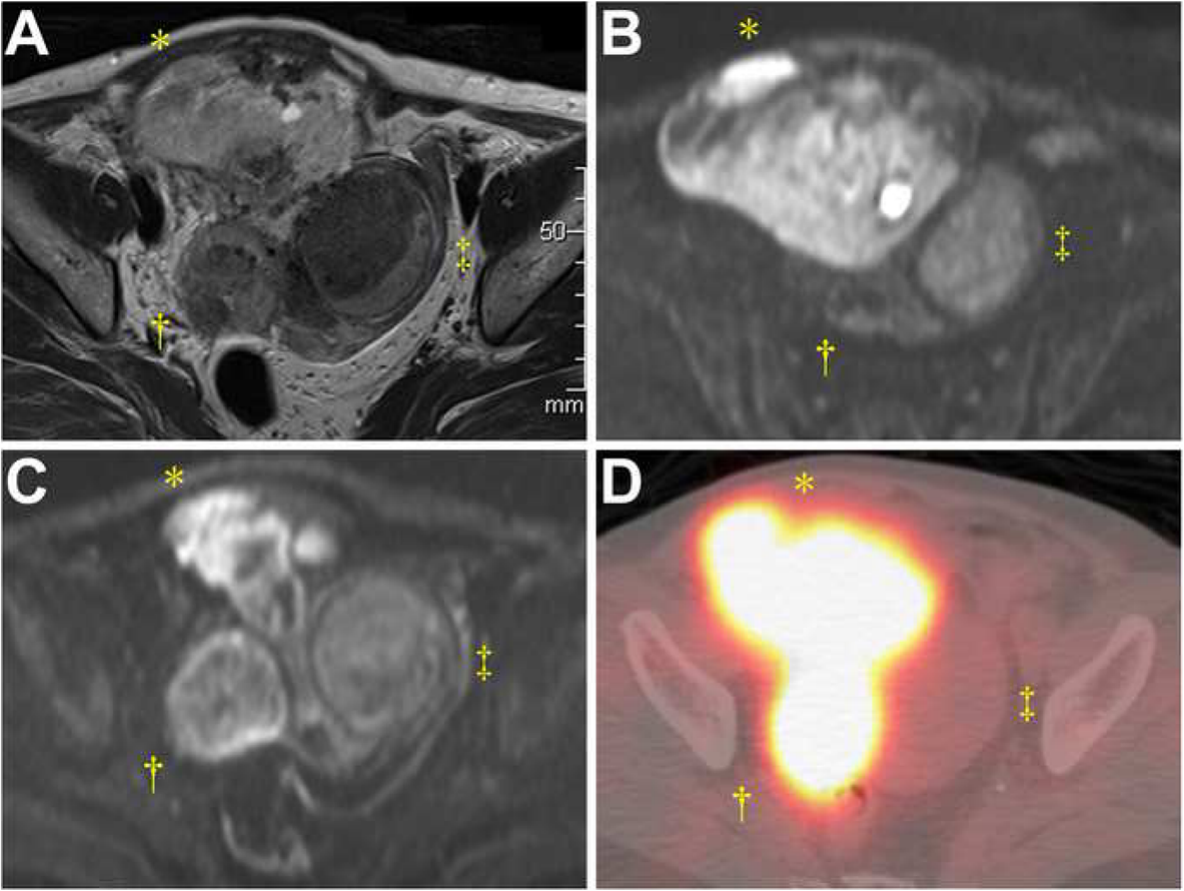
Imaging of the uterine tumor composed of three components. **a** Pelvic axial T2-weighted magnetic resonance imaging disclosing a mass with heterogenous, mixed low and high intensity (*) and another mass with mostly low intensity (†) attached to the right-anterior and right-posterior serosa, respectively, and the remaining low-intensity intramural mass in the fundus (‡). **b** and **c** Diffusion-weighted images showing high intensity in the right-anterior tumor (*) and slightly high intensity in the right-posterior (†) and the fundal (‡) tumors (**d**) Positron emission tomography/computed tomography fusion image demonstrating high ^18^F-fluorodeoxyglucose uptake of the right-anterior and right-posterior tumors (*, †) in contrast to no uptake of the fundal tumor (‡)

A 69-year-old Japanese postmenopausal (gravida 3, para 3) woman went to her primary physician complaining of urinary incontinence 3 months before admission to our institute. She was suspected of having a uterine mass, which had been growing up in size of 8 cm to 9 cm during prior 2 months. Her medical history was unremarkable. She had no past history of neoplasms and no family history of cancer. Blood testing showed slightly elevated levels of lactate dehydrogenase (LDH) 269 U/L (normal range: 124–222 U/L) and cancer antigen (CA) 125 46 U/mL (cutoff value: 35 U/L). CA 19–9 level was within normal limits. The pelvic examination and transvaginal ultrasonography revealed a fist-sized uterine corpus without abnormalities in other reproductive organs. The cervical cytology was normal. Pelvic magnetic resonance imaging demonstrated a 9 × 8 × 7-cm mass protruding from the right-anterior wall of the uterine corpus that showed heterogenous high intensity on T2-weighted images, and high intensity on diffusion-weighted images (DWI), coexisting with a 4.5 × 3.5-cm mass attached to the right-posterior wall of the uterus and a 6.5 × 4.5-cm intramural mass in the fundus, both demonstrating slightly high intensity on DWI (Figs. 1a-c). On ^18^F-FDG-PET, maximum standardized uptake value was 13.28, confined to the tumors located in the uterine right-anterior and right-posterior wall, in sharp contrast to no uptake in the intramural tumor (Fig. 1d). There was no evidence of distant disease. She was radiologically diagnosed with a high-grade uterine sarcoma and concomitant leiomyoma, of which preoperative biopsy was not performed. She underwent total abdominal hysterectomy with bilateral salpingo-oophorectomy and pelvic lymphadenectomy, followed by an uneventful postoperative course. She received no adjuvant therapy, being alive without evidence of recurrence at 14 months after the surgery.

### Pathologic examination

**Fig. 2:**
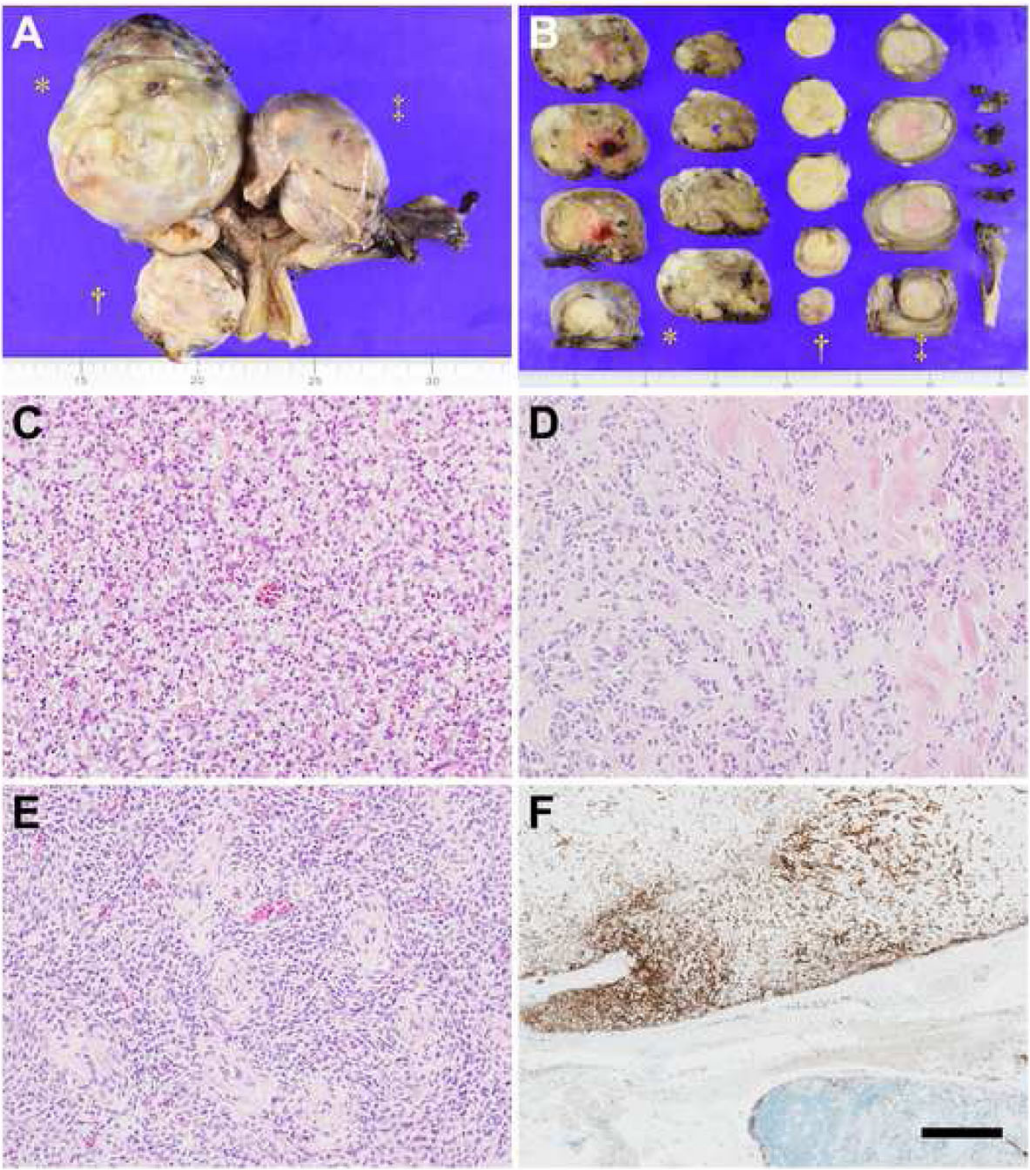
Photographs of the surgically removed uterine tumor. **a** Three uterine masses separately located on the right-anterior (*) and right-posterior (†) serosa and within the fundal myometrium (‡) of 9.5 cm, 4.5 cm, and 6.5 cm in largest diameter, respectively. **b** Cut surfaces of the uterine tumor by lesion, which were heterogenously tan to yellow with focal hemorrhage (*), white with yellow plaques (†), and pale-gray with intratumoral mosaic demarcation (‡). **c**-**e** Cytohistologic variation by lesion. (**c**) Uniform cells having round to ovoid nuclei and scant cytoplasm proliferating with intercellular edema (hematoxylin and eosin [HE] stain, × 100). **d** Tumor cells arranged in trabecular cords admixed with abundant fibromyxoid matrix (HE, × 100). **e** Classic morphology of low-grade endometrial stromal tumor characterized by numerous whorls around small vessels (HE, × 100). **f** Tumor extension into the extrauterine vein positive for CD10 (the upper area), adjacent to nonmetastatic pelvic lymph nodes (immunohistochemical [IHC] stain; bar, 0.5 mm)

Macroscopically, the removed uterus revealed three separate masses, two of which were located on the uterine right-anterior and right-posterior serosa, respectively, with the remaining mass confined to the fundal myometrium (Fig. 2a). Appearances of the cut surfaces varied by lesion, which were yellow to tan or grayish white coloured, solid or lobulated, accompanied by extensive degeneration and focal hemorrhage in the largest tumor (Fig. 2b). On microscopic examination, the three tumors shared the morphologic feature of proliferating cells having round to ovoid nuclei with a high nuclear-to-cytoplasmic ratio, similar to that of proliferative-phase endometrial stromal cells, with somewhat different cytohistologic features including intercellular edema and fibromyxoid changes in the right-anterior and right-posterior tumors, respectively (Figs. 2c-e). Neither round-cell component nor pleomorphism was identified, and mitoses did not exceed 3 counts per 10 high-power-fields (HPF) anywhere in the three tumors. Vascular permeation was prominent and “worm-like” at the periphery of the fundal tumor, accompanied by focal extrauterine extension of the tumor into the pelvis, swelling 2.0 cm in size (Fig. 2f). Neither lymphatic permeation nor lymph-node metastasis was observed. The uterine cervix and both appendages were not involved by the tumor. The histologic diagnosis was Stage IB low-grade ESS according to the WHO and International Federation of Gynecology and Obstetrics 2014 Classification.

### Immunohistochemical study

**Fig. 3:**
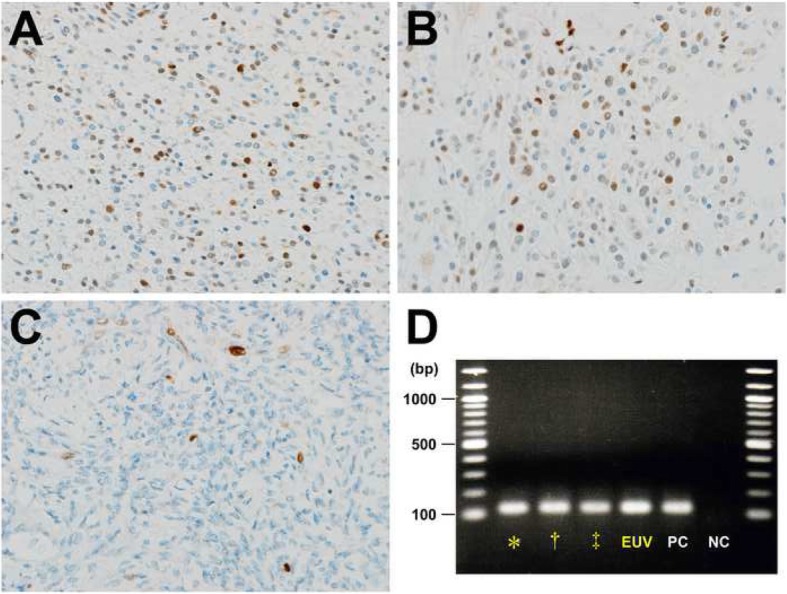
Cyclin D1 immunostains of the endometrial stromal sarcoma harboring the *JAZF1–SUZ12* fusion gene. **a** and **b** 50% nuclear staining of tumor cells for Cyclin D1 in the right-anterior (**a**) and right-posterior (**b**) components (IHC, × 200). **c** Less than 1% of tumor cells positive for Cyclin D1 in the fundal component (IHC, × 200). **d** Reverse transcriptase-polymerase chain reaction demonstrating the *JAZF1–SUZ12* chimeric transcripts in the right-anterior (*), right-posterior (†), and the fundal (‡) tumors and the tumor extension into the extrauterine vein (EUV). PC, positive control; NC, negative control

All the three tumors were positive for CD10, Wilms’ tumor 1, estrogen receptor, progesterone receptor, and androgen receptor in most areas, and focally positive for α-smooth muscle actin and h-caldesmon. Cyclin D1 nuclear staining was positive in 50% of neoplastic cells with weak to moderate intensity in the two subserosal tumors whereas the fundal tumor showed < 5% Cyclin D1 positive tumor cells (Figs. 3a-c). Ki-67 labeling indices of the right-anterior, right-posterior, and fundal tumors were 10, 10, and 3%, respectively.

### Molecular analysis

Reverse transcriptase-polymerase chain reaction (RT-PCR) was performed, using formalin-fixed and paraffin-embedded tissues obtained from each uterine tumor and the extrauterine venous extension. Complementary DNA was produced from 1 μg of RNA using an anchored-Oligo (dT)18 primer and Transcriptor First Strand cDNA Synthesis Kit (Roche Diagnostics, Basel, Switzerland). The RT reaction was performed in total 20 μL at 50 °C for 60 min, followed by heating at 85 °C for 5 minutes. The PCR cycling condition was set as follows; an initial denaturing step at 95 °C for 10 min, and 40 cycles at 95 °C for 1 min, 58 or 60 °C for 1 min,72 °C for 30 s. Primers were as follows: *JAZF1*, forward 5′-AGCAGTGGAAGCCTTACTCC-3′; *SUZ12*, reverse 5′-GCTATGAGATTCCGAGTTCGAAG-3′; *YWHAE*, forward 5′ CACTTATCATGCAGTTGTTACGTGAT-3′; *FAM22*, reverse 5′-GGGCAGAGCCGTGAACAC-3′; β-actin as an internal control, forward 5′- TGGCACCACACCTTCTACAA-3′ and reverse 5′-CCATCACGATGCCAGTGGTA-3′. The same-sized electrophoretic bands indicated amplification of *JAZF1–SUZ12* transcripts (Fig. 3d), although *YWHAE–FAM22* was not detected in all the tumor components.

## Discussion

The t (7;17) translocation resulting in fusion of the *JAZF1* gene (on chromosome 7p15) to the *SUZ12* gene (on 17q21) represents the most common chromosomal rearrangement in low-grade endometrial stromal tumors, being detected in approximately 30% of low-grade ESS and 50% of endometrial stromal nodules tested [4, 5]. Those tumors share characteristic morphologic features of spindle-cell proliferation similar to those of proliferative-phase endometrial stroma, typically lacking significant cytologic atypia and mitotic activity (usually < 5 counts per 10 HPF) [2]. The current WHO Classification of Tumors of Female Genital Organs 2014 reintroduced a terminology of high-grade ESS, which was formerly classified into undifferentiated stromal sarcoma and recently reported to harbor the specific *YWHAE* rearrangement in most cases [6]. High-grade ESS consistently shows uniform cytomorphology, about half of which are biphasic where a round-cell component with mitotic activity (typically > 10 per 10 HPF) is admixed with a low-grade-ESS like spindle-cell component commonly with fibrous/fibromyxoid stroma [7]. Immunohistochemically, the round-cell component usually shows > 70% cyclin D1 nuclear staining of the tumor cells with homogenous moderate to strong intensity, in contrast to the form of scattered positive cells (< 5%) in classic *JAZF1* rearranged endometrial stromal tumors [8]. In the present case, the clinically rapid growing component with high FDG uptake demonstrated around 50% cyclin D1 nuclear immunostaining of neoplastic cells with up to moderate intensity, which indicates the low-grade-ESS like component possibly comprising *YWHAE* rearranged ESS as Lee et al. reported [8]. This paradoxical heterogeneity in a single *JAZF1–SUZ12* ESS with a zonal uptake of FDG has never been documented.

Subserosal masses are much less common in endometrial stromal tumors than in smooth-muscle tumors. The uterine corpus is the most common location for endometrial stromal tumors, although extrauterine locations, specifically the peritoneal surfaces, can be primary sites for the tumor in association with endometriosis, including the ovary, pelvis, fallopian tubes, abdominal cavity, vulva, vagina, bowel wall, urinary bladder, retroperitoneum, and lymph nodes [9, 10]. Recently, Agaimy et al. reported a man with *JAZF1–SUZ12* ESS arising in the paratestis as an extremely rare case [11]. Typically, macroscopic ESS growth patterns feature any combination of (1) intramyometrial nodular masses, (2) an intracavitary polypoid mass, or (3) diffuse myometrial infiltration within expanded uterine walls [2]. The gross appearance of endometrial stromal tumors is occasionally mixed with hemorrhages and necrosis, which may complicate differentiating them from leiomyosarcomas especially when uterine subserosal tumors demonstrate strikingly high FDG uptake. Overt myometrial infiltrations and/or intravascular, worm-like (or “tongue-like”) plugs of tumor protruding from intramyometrial or parametrial veins, which are occasionally recognizable on gross examination, are important in distinguishing ESS from leiomyosarcomas [9]. We speculate the two subserosal masses are metastatic from the intramural lesion based on the identical molecular findings and the presence of venous extrauterine permeation.

There were few reports on FDG-PET findings of ESS histologically examined in a large case series; Yamamoto et al. assessed the mean SUV of ^18^F-FDG-PET of 12 uterine sarcomas (8 leiomyosarcomas, 2 high-grade ESS, and 2 low-grade ESS) at 6.1 ± 3.7 (range, 1.7–13.5; the highest SUV of ESS was 11.70) [12]. Zhao et al. reported the mean SUV of ^18^F-FDG-PET of the largest series of 14 uterine malignancies with a mesenchymal component (8 leiomyosarcomas, 3 carcinosarcomas, 1 ESS, and 2 undifferentiated endometrial sarcomas) to be 5.5 ± 3.1 (range, 1.8–12.0) [13]. To the best of our knowledge in the literature, SUV of 13.28 in the present case is the highest ^18^F-FDG uptake in uterine ESS even though there was no radiologic reason for the exceptional uptake, e.g. coexisting histologic inflammation in tumors. In the present case, preoperative levels of serum CA 125 and LDH were slightly elevated, and the former was normalized postoperatively in contrast to the latter staying at a level around 300 U/L. Therefore, CA 125 may be useful for the diagnosis of uterine sarcomas, although this remains nonspecific due to the possibility of stimulated mesothelial cells.

Low-grade ESS is usually indolent with five-year disease specific survival for stages I and II being 90% compared to 50% for stages III and IV [14]. Patients with high-grade ESS have earlier and more frequent recurrences (often < 1 year) and are more likely to die of disease [7], likely to have an intermediate prognosis between low-grade ESS and undifferentiated uterine sarcoma, the latter of which is usually aggressive and exhibits remarkable pleomorphism with no specific translocation pattern [15, 16]. Since the prognosis of patients with *JAZF1–SUZ12* ESS harboring focally high proliferative activity is still unclear, careful follow-up is recommended and future investigations should target this heterogeneity in a single ESS to clarify its prognostic value.

## Conclusions

High FDG uptake on PET is rare in *JAZF1–SUZ12* ESS, to which the heterogenous proliferative activity in a single ESS may attribute to this extraordinary radiologic finding. We emphasize that pathologists and clinicians should not exclude the possibility of *JAZF1–SUZ12* ESS with a complex growth pattern even when subserosal masses are remarkable, and that molecular analysis is helpful for diagnostic confirmation of *JAZF1–SUZ12* ESS with intrauterine metastasis.

## Data Availability

The datasets used and/or analyzed during the current study are available from the corresponding author upon reasonable request.

## Abbreviations

CA: Cancer antigen
DWI: Diffusion-weighted images
ESS: Endometrial stromal sarcoma
FDG: Fluorodeoxyglucose
HE: Hematoxylin and eosin
IHC: Immunohistochemistry
LDH: Lactate dehydrogenase
MRI: Magnetic resonance imaging
PET: Positron emission tomography
RT-PCR: Reverse transcriptase polymerase chain reaction
WHO: World Health Organization
